# Ocular adverse events associated with eye makeup: a cosmetovigilance-based cross-sectional study of prevalence and predictors among Jordanian women

**DOI:** 10.3389/fpubh.2025.1681656

**Published:** 2025-10-16

**Authors:** Mohammad Abusamak, Sura Al Zoubi, Amal F. Alomari, Sara M. Issa, Ayman A. Abdul Aziz, Asma Musleh, Hala Alrfooh, Yazun Bashir Jarrar, Rasmieh Al-Amer, Roa'a Hamzeh, Lena Al-Kuran, Talal M. Abusamak

**Affiliations:** ^1^Department of Special Surgery, Faculty of Medicine, Al-Balqa Applied University, Al-Salt, Jordan; ^2^Department of Basic Medical Sciences, Faculty of Medicine, Al-Balqa Applied University, Al-Salt, Jordan; ^3^Ministry of Health, Amman, Jordan; ^4^Faculty of Nursing, Al-Yarmouk University, Irbid, Jordan; ^5^The Jordan University Hospital, Amman, Jordan; ^6^AlKasr AlAiny Medical School, Cairo University, Cairo, Egypt

**Keywords:** eye makeup, adverse reactions, cosmetovigilance, Jordanian females, web-based survey, ocular surface

## Abstract

This study aimed to investigate eye makeup adverse reactions (ARs), habits and practices among Jordanian females. The research also sought to identify factors contributing to the risk of ARs to promote safer cosmetic practices and protect public health. A cross-sectional, web-based survey was conducted between March and May 2024, targeting Jordanian female residents. Data was collected using a self-administered questionnaire distributed via social media platforms using snowball sampling. The questionnaire covered social demographic characteristics, eye makeup habits, prevalence of cosmetic ARs, knowledge of eye makeup products, and the Ocular Surface Disease Index (OSDI). Logistic regression was used to assess predictors of eye makeup-related ARs. The study analyzed 1,741 valid surveys. Eye makeup users were generally younger (mean age 29.8 years) than non-users (mean age 36.8 years). A high proportion of eye makeup users (85%) reported at least one AR, with lacrimation being the most common symptom (59.2%). Significant predictors of ARs included young age (AOR = 0.968, *p* = 0.001), food/drug allergies (AOR = 1.602, *p* = 0.005), and allergic ocular disease (AOR = 4.401, *p* < 0.001). Unexpectedly, consistently removing eye makeup before sleep was associated with a higher risk of ARs (AOR = 4.718, *p* = 0.003). In conclusion, this study highlights the prevalence of adverse reactions associated with eye makeup use among Jordanian females and underscores the importance of cosmetovigilance. The high rate of self-reported adverse reactions indicates a need for increased awareness and education regarding safe cosmetic practices. Factors such as young age, pre-existing allergies were identified as significant predictors of adverse events, emphasizing the necessity of targeted interventions.

## 1 Introduction

The use of eye cosmetics is deeply rooted in human history, with archaeological evidence revealing their application in ancient Egypt to enhance physical appearance and convey social identity to both men and women-according to archaeological evidence: cosmetics, in general, have been used since prehistoric times (1–5). This tradition continues today, though modern formulations offer both beauty benefits and potential health risks. In modern times, eye cosmetics are often used to enhance the natural beauty of the eyes and to create an illusion of greater size and magnificence—thus increasing their perceived attractiveness (6–9). In addition, women lavishly spend money on popular cosmetics like eyeliner, mascara, and eyeshades—as an expression of their self-image (3, 5, 6, 10). While cosmetics empower women, they also pose risks of adverse reactions, especially with prolonged use (1, 6, 11).

Eye cosmetics are composed of a variety of substances, such as preservatives, vehicles (agents), antioxidants, humectants, fragrances, ultraviolet absorbers, emollients, emulsifiers, acrylates, and pigments (12, 13). Some of these products may be unsuited for use in close proximity to the eyes and may induce allergic reactions or irritation (12, 14). Likewise, microbial contamination can result from poor handling and storage, and the transmission of infections is further facilitated by the sharing of products (7, 14). Furthermore, the risk of injury increases with the use of aggressive application and removal techniques for instance: the use of fingers or cotton buds to rub eyelashes, which can inflict harm on the fragile ocular surface (15). Building upon the previous information, the risk of adverse reactions also increases with the prolonged use of contact eye makeup products-and these risks are further exacerbated by the lack of consumer awareness regarding ingredient safety, expiration dates, and proper application practices (1, 15, 16).

To address these risks, effective monitoring and regulation through cosmetovigilance is essential for monitoring adverse reactions and ensuring consumer safety (17). Through systematic data collection and analysis of cosmetic-related adverse events, it helps identify risks and implement preventive measures (4, 18, 19). Eye makeup poses particular risks due to factors such as harmful ingredients, poor hygiene, improper application and removal for example the use of fingers without hand washing, prolonged use defined as more than 6 months of regular use of eye makeup, and limited consumer awareness (1, 6, 12, 20).

Despite the wealth of information available globally, limited studies in Jordan have specifically addressed the patterns of cosmetic use among females. There were scarce studies conducted in Jordan in recent years exploring the cosmetics usage patterns and perceptions among females- this could be due to cultural factors or lack of resources. One study investigated skin-lightening products—it was not specific for the ocular area, but no research was published—specifically, on eye makeup use adverse reactions (48). Given the potential risks associated with eye makeup, this study aims to investigate the practices and reported adverse reaction of eye makeup use among Jordanian females. More specific aims: it seeks to determine the prevalence and nature of adverse reactions experienced from an ophthalmological point of view, and whether it is influenced by age, education level, or other socioeconomic factors. The findings of this research may contribute to a deeper understanding of the challenges and opportunities in promoting safer cosmetic guidelines and enhancing community awareness and protection.

## 2 Methods and results

### 2.1 Study design and ethics approval

A cross-sectional web-based survey was conducted between March and May 2024 to explore eye makeup practices and related adverse effects among women in Jordan. Data were collected via a self-administered questionnaire distributed through social media platforms. The study protocol received ethical approval from the Institutional Review Board of Al-Balqa Applied University (Ref No: 55/2023. Date 22/11/2023) and complied with the Declaration of Helsinki (2013) (21). Participation was voluntary and anonymous. Informed electronic consent was obtained prior to accessing the survey questions.

### 2.2 Study population and sampling

Eligible participants were female residents of Jordan aged 18 years or older. A non-probability sampling approach was employed, combining convenience and exponential non-discriminative snowball sampling. The questionnaire link was disseminated via university student forums and women's social media groups across various Jordanian governorates. Participants were encouraged to share the link within their networks to enhance reach. Eligibility was confirmed through screening questions embedded in the survey. Jordan has a population of approximately 11,734,000 people, with a median age of 22.9 years, with women accounting for 47.1% (22).

### 2.3 Survey instrument

The questionnaire was developed following a review of relevant literature and comprised seven sections (23–29): **Introduction and Consent**—including objectives, definitions of eye makeup, a confidentiality statement to ensure user privacy, and right to withdraw at anytime. Second section discussed the **Eligibility Screening**—two questions on gender and age; directing respondents to exit the survey if they did not meet the inclusion criteria of being female and older than 18 years old. Third section was on **Sociodemographic Profile** that included 14 items including age, education, marital status, occupation, smoking history, digital device use, and allergy history. Fourth part of the questionnaire focused on **Makeup Practices**— 18 items assessing frequency, method of application and removal, duration of use, and product expenditure. Fifty section inquired about the **Adverse Reactions of using eye makeup consisted of** 21 items covering symptoms such as eye redness, swelling, loss of eyelashes, abrasions of the cornea, seborrheic blepharitis, eye bibles, dermatitis, eye discharge, itchiness, etc. The sixth section was about the **Knowledge Assessment** of participants in the format of 10 questions on eye makeup ingredients, expiration dates, and labeling, with a scoring system. The last section consisted of the Ocular Surface Disease Index (OSDI) scale of 12 standardized questions in the Arabic language (30).

### 2.4 Development of the questionnaire

The majority of the questions in this study were extracted from previous research—please refer to previous paragraph, which was written in simple Arabic. Some questions required both forward and backward translations. Furthermore, the authors self-developed the questions, tested them for face and content validity, and made necessary amendments.

### 2.5 Pilot testing

A pilot study was carried out 1 month before distribution of the questionnaire to evaluate item clarity and content validity. Several face-to-face interviews were performed to collect participant feedback on the questionnaire's structure and wording. Based on this feedback, no substantive modifications were required for the final instrument. Data from pilot participants were not included in the primary analysis.

### 2.6 Sample size

Sample size was estimated using the formula *n* = 100 + 50*i*, where *i* is the number of independent variables. In general, a sample size of around 10% of the population, but not exceeding 1,000 participants, was considered appropriate. We used an event per variable (EPV) of 50 and the formula *n* = 100 + 50*i*, where *i* represents the number of independent variables in the final model. In our study, the minimum sample size would be 850, as we conducted logistic regression for 15 predictors (31). Although the intended upper limit was 1,000, a total of 1,837 responses were received, of which 1,741 met eligibility criteria and were included in the final analysis.

### 2.7 Data management and statistical analysis

Data were collected via Google Forms^®^, cleaned to remove incomplete or invalid responses (e.g., age < 18 or >90, non-differentiated responses), and exported to Google Sheets^®^ for de-identification. Final datasets were imported into STATA SE v14 (2015) for analysis (32). Descriptive statistics summarized demographics, usage patterns, and reported symptoms. Bivariate associations were explored using Chi-square tests. Logistic regression analysis was conducted to identify predictors of self-reported adverse events. Variables significant in bivariate analysis were included in multivariate models. Both crude and adjusted odds ratios (COR, AOR) with 95% confidence intervals were reported. Significance threshold was set at p < .05 as appropriate.

### 2.8 Operational definitions and variables

**Adverse reactions** were defined as any ocular symptom perceived by the respondent to be linked to eye makeup use (26). **Eye makeup** referred to any cosmetic or applicator used on or around the eye and eyelids; articles that include perfumes, skin care, personal care, or hair care on the face or other body parts were excluded from the definition (3, 26). The primary outcome was the presence of one or more self-reported adverse effects. Independent variables included demographic factors, product usage behaviors, and hygiene practices.

## 3 Results

### 3.1 Descriptive statistics of the study population

Among 1,741 valid surveys, eye makeup users (*n* = 1,438) were significantly younger than non-users (mean age: 29.8 ± 9.8 vs. 36.8 ± 12.6 years, *p* < 0.001) and more likely to be single (56.1%), university-educated (74.0%), and employed in office-based (13.1%) or health-related roles (18.0%) (*p* < 0.001). They reported higher rates of smoking (26.6%), contact lens use (14.1%), oral acne medication (28.5%), and food/drug allergies (17.8%) compared to non-users (all *p* < 0.05). Chronic eye disease was more prevalent among non-users (4.0% vs. 1.5%, *p* = 0.006), as demonstrated in Table 1.

**Table 1:** Sociodemographic characteristics of participants (*N* = 1,741).

**Variables**	**Categories**	**Use of eye makeup**			***X* ^2^**	***P*-value**
		**No** ***n*** **(%)**	**Yes** ***n*** **(%)**	**All** ***n*** **(%)**		
Age (in years) [mean, (SD), range]		36.8 (12.6), (18–70), *n =* 303	29.8 (9.8), (18–68), *n =* 1438	31.1 (10.7), 18–68	217.9	< .001
Age	18–23	70 (23.1)	484 (33.7)	554 (31.8)	123.85	< 0.001
	24–39	77 (25.4)	656 (45.6)	733 (942.1)		
	>39	156 (51.5)	298 (20.7)	454 (26.1)		
Education level	Secondary school	27 (8.9)	106 (7.4)	133 (7.6)	14.97	0.0018
	Community College	29 (9.6)	89 (6.2)	118 (6.8)		
	University	192 (63.4)	1,064 (74.0)	1,256 (72.1)		
	Post graduate	55 (18.1)	179 (12.4)	234 (13.4)		
Occupation	Student	80 (26.4)	421 (29.3)	501 (28.8)	45.34	< 0.001
	Academic	88 (29.0)	225 (15.7)	313 (18.0)		
	Health Worker	40 (13.2)	259 (18.0)	299 (17.2)		
	Field Worker	6 (2.0)	49 (3.4)	55 (3.2)		
	Office Worker	16 (5.3)	189 (13.1)	205 (11.8)		
	Unemployed	73 (24.1)	295 (20.5)	368 (21.0)		
Smoking	No	277 (91.4)	1,056 (73.4)	1,333 (76.6)	45.11	< 0.001
	Yes	26 (8.6)	382 (26.6)	408 (23.4)		
Social status	Single	117 (38.6)	806 (56.1)	923 (53.0)	33.08	< 0.001
	Married	165 (54.5)	582 (40.5)	747 (43.0)		
	Other	21 (6.9)	50 (3.4)	71 (4.0)		
Are you a health worker/student?	No	189 (62.4)	886 (61.6)	1,075 (61.8)	0.06	0.8039
	Yes	114 (37.6)	552 (38.4)	666 (38.2)		
Chronic eye disease	No	291 (96.0)	1,416 (98.5)	1,707 (98.0)	7.72	0.0055
	Yes	12 (4.0)	22 (1.5)	34 (2.0)		
Allergic eye diseases	No	258 (85.2)	1,208 (84.0)	1,466 (84.2)	0.25	0.62
	Yes	45 (14.8)	230 (16.0)	275 (15.8)		
Contact lenses	No	284 (93.7)	1,235 (85.9)	1,519 (87.3)	13.85	0.0002
	Yes	19 (6.3)	203 (14.1)	222 (12.8)		
Any eye surgery	No	272 (89.8)	1,237 (86.0)	1,509 (86.7)	3.04	0.0811
	Yes	31 (10.2)	201 (14.0)	232 (13.3)		
Oral acne medications	No	261 (86.1)	1,028 (71.5)	1,289 (74.0)	27.95	< .001
	Yes	42 (13.9)	410 (28.5)	452 (26.0)		
Food drug allergy	No	267 (88.1)	1,181 (82.1)	1,448 (83.2)	6.42	0.0113
	Yes	36 (11.9)	257 (17.8)	293 (16.8)		

### 3.2 Characteristics, practices, and habits of eye makeup users

**Figure 1:**
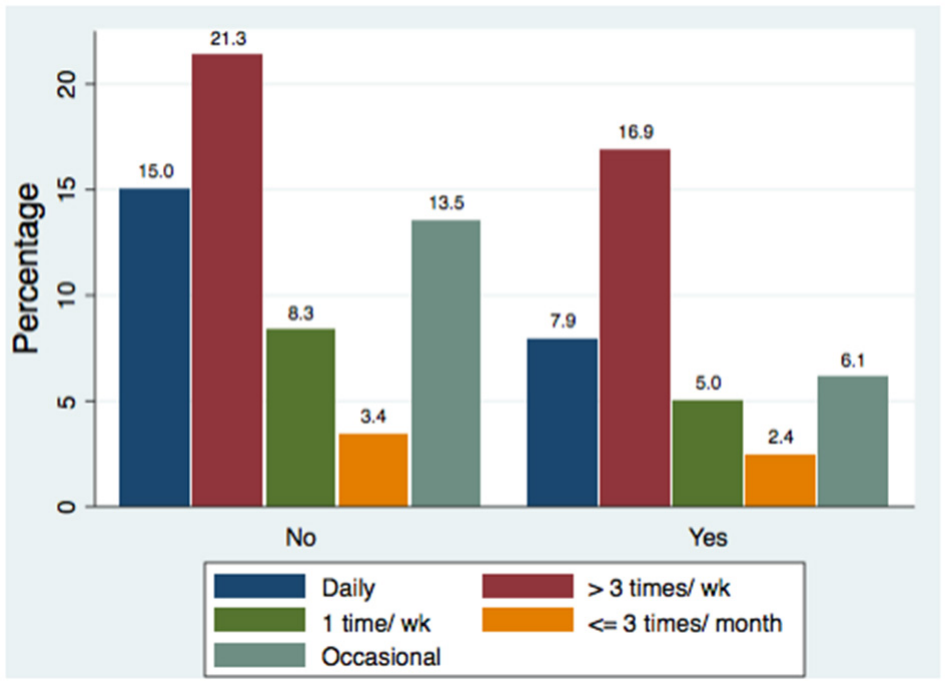
Eye makeup frequency by study of health disciplines.

Table 2 illustrates that among 1,438 eye makeup users, the mean age of first use was 19.7 ± 5.7 years, with an average duration of use of 10.1 ± 7.6 years. Most eye makeup users applied makeup at least three times per week and spent less than 50 JOD per month. Spending was significantly associated with the employment category (χ^2^ = 32.61, *p* = 0.005), with healthcare and field workers spending more than unemployed participants. Most users applied makeup themselves using brushes or fingers and based purchasing decisions on brand and color. Specialist stores and online retailers were the preferred sources. A considerable proportion ignored product expiration dates. Micellar water and soap-based cleansers were the most common removers. Participants in health-related fields applied eye makeup less frequently than those outside such fields (Figure 1), especially among students.

**Table 2:** Demographic and socio-economic factors of eye makeup users.

**Characteristic**	**Category**	**Frequency**	**Percentage (%)**
Age [Mean ± SD, (Range)]	Users *n =* 1,438	29.9 ± 9.9, (18, 68)	
	Non-users *n* = 303	36.6 ± 12.7, (18,64)	
Years using eye makeup	Users *n =* 1.438	10.1 ± 7.6, (1,45)	
Age begin using eye makeup	Users *n =* 1.438	19.7 ± 5.7, (4,59)	
What is the frequency of eye makeup usage?	Daily	330	22.95
	>3 times/week	550	38.25
	1 time/week	192	13.35
	≤ 3 times/month	84	5.84
	Occasional	282	19.61
Money expenditure on buying Eye Makeup	< 10 JOD	802	55.77
	10–50 JOD	547	38.04
	50–100 JOD	71	4.94
	>100 JOD	18	1.25
How do you apply eye makeup	Myself	1,392	96.8
	Others	46	3.2
Why do you continue to keep eye makeup products?	Cost	429	29.8
	No Alternative color	392	27.3
	Don't know if expired	617	42.9
What is the most important factor when you buy a certain product?	Brand	627	43.6
	Price	216	15.02
	Color	595	41.38
Where do you buy eye makeup products? More than one choice	Specialty store	1,054	
	Pharmacy	217	
	Online-shop	332	
	Abroad	281	
	General store	468	
Eye makeup applicator preferences: more than one choice	Brush	1,088	
	Single use applicator	135	
	Sponge	489	
	Cotton Buds	331	
	Finger application	545	
Removal method makeup	Water	73	5.08
	Soap Water	318	22.11
	Micellar	511	35.54
	Foam	75	5.22
	Cream	247	17.18
	Lotion	39	3
	Wipes	172	11.96
	Never	3	0.21

Note Some percentages for multiple-choice questions may exceed 100% due to participants selecting more than one option. Values are presented as n (%) unless otherwise specified.

### 3.3 Safety and Risk practices of participants

In general, hygiene practices were suboptimal, as demonstrated in Table 3. Although 89.4% of participants reported washing their hands before application and 58.2% removed makeup before sleep, it was common for them to share products, skip skin patch tests, and neglect ingredient lists. Only 19.1% checked expiration dates regularly.

**Table 3:** Safety behaviors of eye cosmetics habits (*n* = 1,148).

**Question**	**Response**	**Frequency (n)**	**Percentage (%)**
Hand washing before application	Never	153	10.64
	Sometimes	539	37.48
	Always	746	51.88
Share makeup with others	Never	578	40.19
	Family	743	51.67
	Anyone	117	8.14
Use testers before purchase	No	1,164	80.95
	Yes	274	19.05
Reason for discarding eye makeup	Expired	297	20.65
	Bottle empty	785	54.59
	When get bored	99	6.88
	Regularly	67	4.66
	If spoiled	162	11.27
	I keep it	28	1.95
Check ingredients before buying	No	1,080	75.1
	Yes	358	24.9
Patch test before use	No	1,147	79.76
	Yes	291	20.24
Clean applicators—how often?	Everytime	137	9.53
	Few Times	691	48.05
	Monthly	337	23.44
	Annually	80	5.56
	Never	193	13.42
Remove makeup before bed	Never	22	1.53
	Sometimes	578	40.19
	All the time	838	58.28

**Figure 2:**
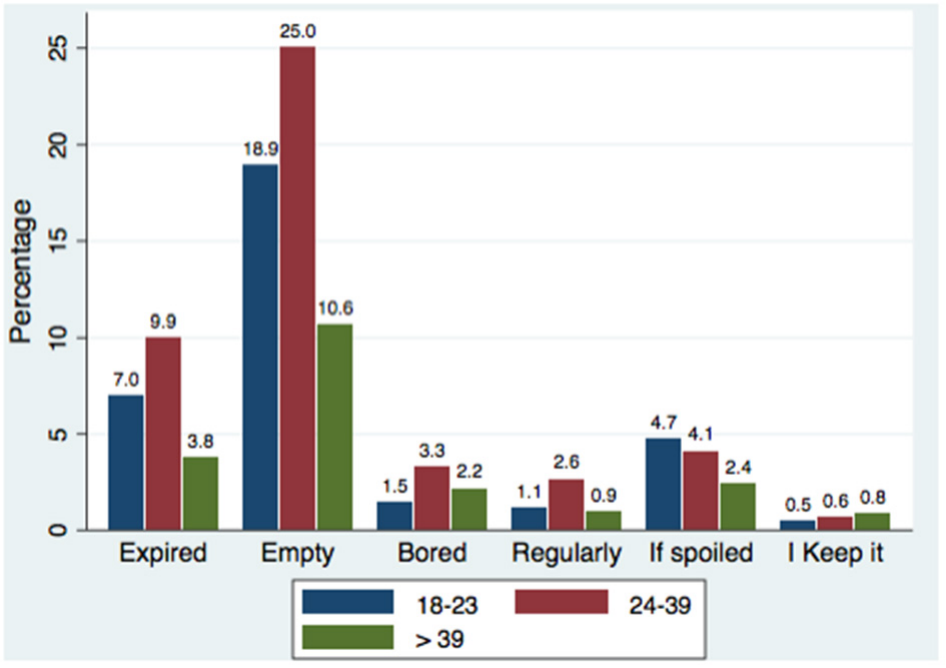
Reasons for discarding eye makeup products according to age groups.

Product disposal behaviors varied by age (χ^2^ = 30.67, *p* = 0.001). Across all age groups, “product empty” was the most cited reason (54.6%). Disposal due to expiration was more common with increasing age (33.7% in 18–23 vs. 48.2% in 24–39 years). Older participants showed higher rates of discarding products due to disinterest/boredom (10.4%), while younger users more often cited spoilage (Figure 2).

### 3.4 Adverse reactions reported by eye makeup users

Among users, 59.2% reported lacrimation, 47.5% dry/foreign body sensation, 43.7% eyelash loss, and 39.4% burning sensation. Other symptoms included itching (38.4%), conjunctival hyperemia (36.2%), brittle lashes (48.0%), eye pain (24.4%), and blurred vision (25.0%). Corneal abrasion was reported by 6.2% of users, while 15.7% experienced chemical irritation. Skin rash around the eyes was reported in 16.97% (Table 4).

**Table 4:** Adverse reactions to Eye makeup products in the last month.

**A. Symptoms after applying eye makeup**	**Never**	**1–3 times**	**>3 times**	**Total adverse events**
Skin erythema around the eyes	1,194 (83.03)	182 (12.66)	62 (4.31)	244 (16.97)
Skin itchiness	980 (68.15)	360 (25.03)	98 (6.82)	458 (31.85)
Skin swelling	1,155 (80.3)	215 (15.0)	68 (4.7)	283 (19.7)
Lacrimation	586 (40.8)	571 (39.7)	281 (19.5)	852 (59.2)
Conjunctival redness	917 (63.8)	380 (26.4)	141 (9.8)	521 (36.2)
FB/dry eye sensation	756 (52.6)	464 (32.3)	218 (15.2)	682 (47.5)
Burning sensation	872 (60.6)	412 (28.7)	154 (10.7)	566 (39.4)
**B. Symptoms related to margin and eyelashes**	**Never**	**1–3 times**	>**3 times**	**Total adverse events**
Blurred vision after internal eyeliner/Kohl	1,079 (75.0)	294 (20.5)	65 (4.5)	359 (25)
Brittle eyelashes	744 (52.0)	444 (30.9)	246 (17.1)	690 (48.0)
Loss of eyelashes (Madarosis)	810 (56.3)	448 (31.2)	180 (12.5)	628 (43.7)
Seborrhea of eyelashes	1,187 (82.6)	178 (12.4)	73 (5.0)	251 (17.4)
Recurrent pimples (styes)	1,201 (83.5)	188 (13.0)	49 (3.4)	237 (16.4)
Eye pain after applying mascara eye lashes	1,087 (75.6)	274 (19.0)	77 (5.4)	351 (24.4)
**C. Symptoms related to injury**	**Never**	**1–3 times**	> **3 times**	**Total adverse events**
Corneal abrasion required medical attention	1,348 (93.7)	71 (4.9)	19 (1.3)	90 (6.2)
Eyelid redness (chemical irritation-removal)	1,212 (84.3)	170 (11.8)	56 (3.9)	226 (15.7)
Itching-Chemical removal of eye makeup	885 (61.5)	436 (30.3)	117 (8.1)	553 (38.4)
Lacrimation-Chemical	923 (64.2)	379 (26.4)	136 (9.4)	515 (35.8)
Eye discharge	1,208 (84.0)	180 (12.5)	50 (3.5)	230 (16.0)

### 3.5 Predictors of occurrence of adverse reactions

**Figure 3:**
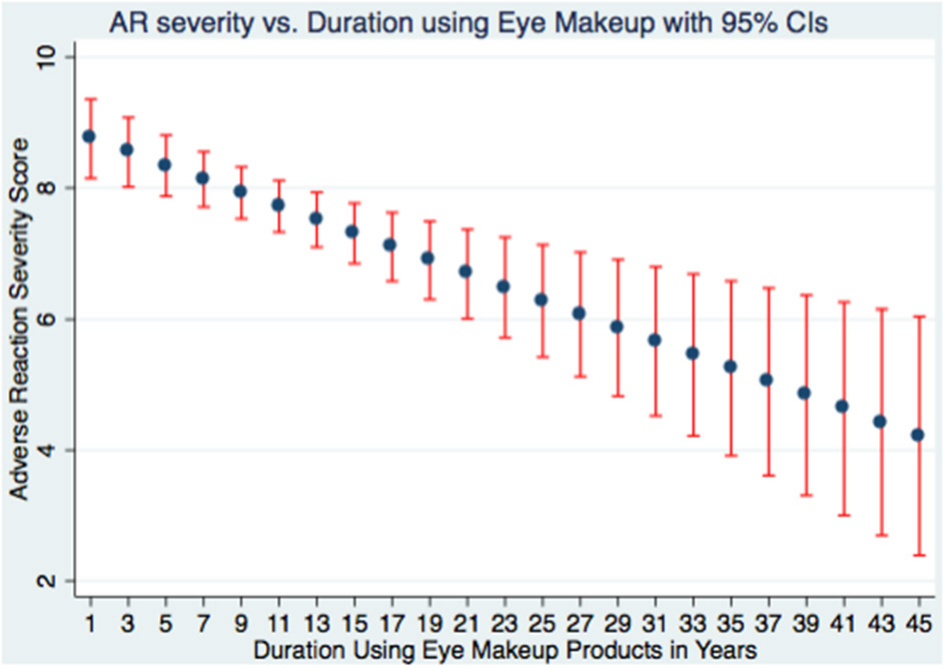
A plot of the adverse reactions score vs. the duration using eye makeup.

**Figure 4:**
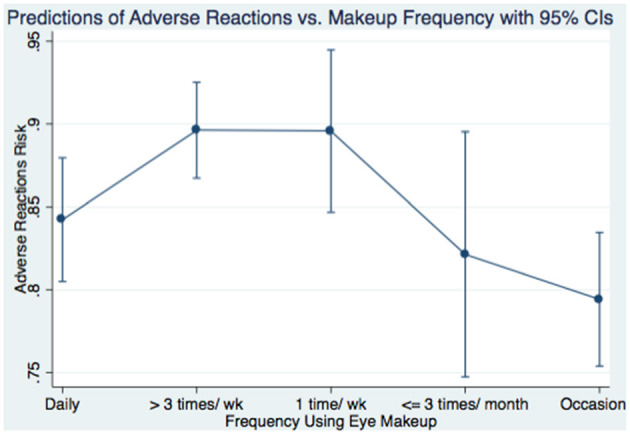
Predictions of adverse reactions score vs. the frequency of using eye makeup products.

Multivariable logistic regression identified significant predictors of adverse reactions (Table 5). Younger age was protective (AOR = 0.968, *p* = 0.001). History of food/drug allergy (AOR = 1.602, *p* = 0.005) and allergic eye disease (AOR = 4.401, *p* < 0.001) increased risk. Unexpectedly, removing makeup before sleep was associated with greater odds of adverse reactions (AOR = 4.718, *p* = 0.003). Use of tester products also increased risk (AOR = 1.77, *p* = 0.016). Other behavioral and sociodemographic factors did not reach statistical significance. A regression plot (Figure 3) showed adverse reaction scores decreased with increasing years of use. Higher frequency of use was associated with greater adverse scores (Figure 4).

**Table 5:** Predictors of adverse reactions to eye makeup products *n* = 1,438.

**Variables (predictors)**		**Observations**	**Percentage**	**COR [CI 95%]1**	**AOR [95% CI]1**	***p*-value^a^**
Age		1,438		0.9758 (0.962, 0.9890)^***^	0.968 (0.949, 0.989)^***^	< .001
Social Status	Ref (Single)	994	57.1			
	Married	747	42.9	0.904 (0.669, 1.222)	1.158 (0.758, 1.768)	0.277
Education	Ref (Secondary or Less)	133	7.6			
	College or More	1,608	92.4	1.095 (0.63, 1.906)	1.334 (0.714, 2.492)	0.365
Study health sciences	Ref (No)	1,075	61.7			
	Yes	666	38.3	0.877 (0.648, 1.187)	0.712 (0.462, 1.097)	0.123
Occupation	Ref (Student)	501	28.8			
	Academic	313	18.0	0.716 (0.454, 1.13)	0.987 (0.528, 1.844)	0.967
	Health	299	17.2	0.772 (0.495, 1.204)	1.177 (0.69, 2.007)	0.550
	Field Work	55	3.2	1.24 (0.47, 3.27)	1.479 (0.526, 4.155)	0.458
	Office Work	205	11.8	0.718 (0.443, 1.163)	0.839 (0.465, 1.511)	0.558
	Unemployed	368	21.1	0.983 (0.626, 1.542)	1.53 (0.833, 2.81)	0.170
Food drug allergy	Ref (No)	1,448	83.2			
	Yes	293	16.8	1.921 (1.207, 3.058)^**^	1.602 (1.152, 2.228)^***^	0.005
Allergic eye disease	Ref (No)	1,466	84.2			
	Yes	275	15.8	3.739 (2.001, 6.986)^***^	4.401 (2.304, 8.408)^***^	< .001
Monthly spending (JOD)	Ref (< 10 JOD)	802	55.8			
	10–50	547	38.0	1.541 (1.113, 2.135)^*^	1.287 (0.904, 1.833)	0.162
	50–100	71	4.9	1.332 (0.646, 2.749)	1.243 (0.583, 2.650)	0.574
	>100	18	1.3	1.547 (0.352, 6.812)	1.459 (0.311, 6.834)	0.632
Frequency using eye makeup	Ref (Occasional)	558	38.8			
	Frequent	880	61.2	1.415 (1.048, 1.909)^**^	1.321 (0.952, 1.833)^*^	0.096
Hand sanitation	Ref (Never)	153	10.6			
	Always	1,285	89.4	1.29 (0.822, 2.025)	1.539 (0.941, 2.519)^*^	0.086
Share eye makeup	Ref (Never)	578	40.2			
	Always Share it	860	59.8	1.555 (1.153, 2.096)^**^	1.334 (0.965, 1.845)^*^	0.082
Use eye makeup testers	Ref (No)	1,164	81			
	Yes	274	19	1.88 (1.201, 2.944)^**^	1.77 (1.112, 2.817)^**^	0.016
Read composition of products	Ref (No)	1,080	75.1			
	Yes	358	24.9	0.752 (0.541, 1.045)^*^	0.87 (0.584, 1.295)	0.492
Patch skin for allergy	Ref (No)	1,147	79.8			
	Yes	291	20.2	0.897 (0.624, 1.288)	0.908 (0.592, 1.393)	0.658
Clean eye makeup applicators	Ref (Rarely)	273	19			
	Monthly	1,028	71.5	1.449 (1.007, 2.086)^*^	1.454 (0.971, 2.179)^*^	0.069
	Always	137	9.5	0.888 (0.523, 1.509)	1.039 (0.567, 1.904)	0.901
Remove makeup before sleep	Ref (Rarely)	578	40.8			
	Always Remove it	838	59.2	0.707 (0.517, 0.967)^**^	4.718 (1.693, 13.151)^**^	0.003

^***^p < 0.01, ^**^p < 0.05, ^*^p < 0.1.

^a^Crude and Adjusted odds ratio was reported using multivariate logistic regression.

### 3.6 Predictors of malpractice habits vs. independent variables

A composite malpractice score was modeled against demographic and behavioral variables (Table 6). This score is based on the following malpractice habits that include not washing hands, sharing makeup, using tester products, not disposing of products regularly, not checking ingredients, not conducting skin patch tests, not cleaning applicators or brushes, and not removing makeup before bed as a single malpractice score value. We found that younger age groups (β = 0.065, *p* < 0.001) and longer duration of use (β = 0.455, *p* < 0.001) predicted higher malpractice scoring. Being married (β = 3.986, *p* = 0.005) and having higher education (β = 7.484, *p* < 0.001) were also associated with poorer safety practices. In contrast, frequent users had significantly lower malpractice scores (β = −15.441, *p* = 0.001), suggesting better hygiene awareness among this subgroup. Although the logistic regression model's overall explanatory power was limited, we demonstrated that multiple factors may play a role in malpractice scores related to eye makeup.

**Table 6:** Predictors of malpractice with independent variable (*n* = 1,741).

**Variables**		**Observations**	**Percentage**	**Coefficient (C.I. 95%)**	***P*-value**
Age in years		1,438		0.065 (−0.1, 0.231)	< 0.001^***^
Years using eye makeup		1,438		0.455 (0.226, 0.685)	< 0.001^***^
Age at begin use eye makeup		1,438		−0.455 (−0.685, −0.226)	< 0.001^***^
Social status	Ref (Single)	856	59.50		
	Married	582	40.50	3.986 (1.196, 6.775)	0.005^***^
Education	Ref (Secondary or Less)	106	7.40		
	College or More	1,332	92.60	7.484 (3.437, 11.531)	< 0.001^***^
Study health sciences	Ref (No)	886	61.6		
	Yes	552	38.4	−0.883 (-3.646, 1.879)	0.531
Smoking	Ref (No)	1,056	73.4		
	Yes	382	26.6	1.183 (-1.174, 3.54)	0.325
Occupation	Ref (Student)	421	29.3		
	Academic	225	15.7	−4.556 (−8.583, −0.528)	0.027^**^
	Health	259	18	0.296 (−3.208, 3.801)	0.868
	Field Work	49	3.4	−2.272 (−8.134, 3.59)	0.447
	Office Work	189	13.1	−3.752 (−7.56, 0.055)	0.053^*^
	Unemployed	295	20.5	−3.296 (−7.003, 0.412)	0.081^*^
Food drug allergy	Ref (No)	1,181	82.1		
	Yes	257	17.9	3.643 (1.474, 5.812)	0.041^**^
Allergic eye disease	Ref (No)	1,208	84.0		
	Yes	230	16.0	−4.214 (−6.375, −2.053)	0.777
Frequency using eye makeup	Ref (Occasional)	558	38.8	−11.54 (−16.25, −6.83)	
	Frequent	880	61.2	−15.441 (−24.5, −6.383)	0.001^***^
Monthly spending (JOD)	Ref (< 10JOD)	802	55.8		
	10–50 JOD	547	38.0	−2.745 (−5.375, −0.115)	< 0.001^***^
	50–100 JOD	71	4.9		< 0.001^***^
	>100 JOD	18	1.3	−0.396 (−3.137, 2.346)	0.001^***^
History of oral acne medication	Ref (No)	1028	71.5		
	Yes	410	28.5	−0.592 (−2.839, 1.655)	0.605
Mean dependent var.		46	SD dependent var.		19.663
*R*-squared		0	Number of obs.		1,438
*F*-test		5	Prob > *F*		< 0.001^***^
Akaike crit. (AIC)		12,588	Bayesian crit. (BIC)		12,698.764

^***^p < 0.01, ^**^p < 0.05, ^*^p < 0.1.

## 4 Discussion

This study showed that women who use eye makeup are significantly younger (mean age 29.8 ± 9.8 years) than non-users (36.8 ± 12.6 years), consistent with prior literature reporting higher cosmetic usage among younger age groups (2, 13, 33). Park et al. (13) found peak usage of eye and nail cosmetics among Korean women aged 20–29, which aligns with our age-stratified findings. Eye makeup users were also more likely to be single, university-educated, and employed in office or health-related sectors, mirroring demographic trends observed in similar populations (6).

Despite a lower prevalence of chronic eye diseases among users, they demonstrated higher rates of contact lens wear, smoking, oral acne medication use, and reported food or drug allergies (*p* < 0.001). These comorbidities may predispose individuals to heightened ocular sensitivity or increased susceptibility to cosmetic-related adverse reactions (12, 16, 34–36).

Regular cosmetic use, defined as ≥3 applications per week, was reported by 61.2% of respondents; in line with the 58.8% observed by (6), where 58.8% were classified as regular users (6). Interestingly, we observed an inverse correlation between usage frequency and adverse reaction scores (Figure 4), suggesting improved hygiene, adaptive tolerance, or informed product selection over time (17, 18). Several factors may explain this: (1) lower malpractice scores among regular users indicate better hygiene practices; (2) repeated exposure may induce ocular tolerance; and (3) experienced users may preferentially select less irritant products and utilize gentler removal techniques (1, 8). Furthermore, our data show an inverse relationship between years of cosmetic use and adverse symptom scores (Figure 3), indicating potential acclimation or avoidance of irritant products (8). Additionally, underreporting among long-term users due to recall bias or normalization of symptoms may play a role (26, 33).

We also noted behavioral variations in makeup removal practices by age and discipline. Among users aged 18–39, 61% preferred micellar or soapy water. In contrast, 51% of women over 39 favored creams or soaps. Health-related discipline participants reported higher usage of micellar water (47.75%) and lower reliance on potentially irritating wipes and foams (31.98% and 29.96%, respectively). This may reflect increased awareness of dermatologic safety and environmental impact. These trends are consistent with previous reports emphasizing the role of cleansing agents in maintaining periocular health (1, 37). Ozdemir et al. (38) similarly reported that although 77.7% of students prioritized makeup removal, only 46.9% used appropriate cleansing agents. Alarmingly, 66.8% of non-health discipline students in our cohort reported never removing makeup before sleep, a potential risk factor for ocular surface inflammation (1). This hygiene gap highlights the need for targeted education, particularly outside the health sciences.

Analysis of the relationship between participants' monthly expenditure on eye makeup products revealed that unemployed women in Jordan, expectedly, were more likely to spend the least amount (less than 10 JOD), whereas professionals in health and fieldwork were slightly more inclined to spend moderately (10–50 JOD) (χ^2^ = 32.6148, *p* = 0.005). The three most common methods of buying cosmetic products included specialty stores, general stores, and online shopping. Comparatively, Meharie et al. (24) reported that the primary source of cosmetics for female students at the Dessie campus, Wollo University in Ethiopia was local or ordinary shops (88.8%), followed by supermarkets (45.8%) and drug retail outlets (24.8%). Their findings also indicated a statistically significant association between monthly income and cosmetics utilization (OR = 2.280, 95% CI = 1.169–7.638) (24). They also reported that cosmetics utilization increased approximately twofold among students with a monthly income of 500 birr (equals 3.88 USD) or more compared to those earning less than 500 birr per month. Several other studies have suggested that income and access to different retail locations can influence cosmetic purchasing habits and utilization (1, 17, 23, 24, 29).

Our findings show that both the field of work and the total amount of disposable income a person has influence purchasing behavior and product choice. For instance, unemployed women were more likely to spend less than 10 JOD per month, whereas health and field professionals were much more inclined to spend between 10 and 50 JOD per month (χ^2^ = 32.6148, *p* = 0.005). These findings mirror those of Meharie et al. (24), who found that Ethiopian students who made more money were more likely to use cosmetics (OR = 2.28, 95% CI: 1.17–7.64). Several previous studies have reported that income and access to different retail locations can influence cosmetic purchasing habits and utilization (1, 17, 23, 24, 29).

Despite the widespread availability of cosmetic products, awareness of product safety remains suboptimal. In our sample, 75% did not read ingredient labels, and 79.8% did not perform skin patch testing prior to use. These findings are consistent with Meharie et al. (24), who found that 66.8% of users overlooked expiration labels. Dibaba et al. (17) and Nayak et al. (11) also noted low engagement in precautionary practices (23). Addis et al. (1) observed that users who routinely read labels had lower rates of adverse reactions. This emphasizes the need for targeted consumer education campaigns to improve safety practices, particularly among younger or less health-literate groups.

In this study, a substantial proportion of eye makeup users (85%) reported experiencing at least one adverse reaction (11, 17, 18, 23, 24, 26). The most frequently reported symptom was lacrimation, affecting 59.2% of users. This symptom, while often dismissed as mild, is clinically significant, as excessive tearing may reflect ocular surface irritation or early evaporative dry eye disease (DED) (2, 10). The second and third most common symptoms—foreign body sensation (47.5%) and burning sensation (39.4%)—are hallmark indicators of tear film instability and ocular surface inflammation (1–3, 18). These symptoms align with previous studies linking frequent cosmetic use, particularly of eyeliner and mascara, to dry eye symptomatology and meibomian gland dysfunction (3, 8, 39).

Hunter et al. (39) further demonstrated that certain eye cosmetics alter the biophysical properties of meibum, increasing its viscosity and contributing to evaporative dry eye. This aligns with our data showing that dry eye symptoms—particularly foreign body sensation and burning—were prevalent, especially among long-term users. These physiological changes reinforce the notion that product selection and application technique are central to ocular surface health in cosmetic users.

The anatomical proximity of eye makeup application to the lid margin and tear film puts the ocular surface at unique risk. Products such as pencil eyeliner, often applied directly to the waterline (mucocutaneous junction), are especially concerning. Albarrán et al. (40) demonstrated that cosmetic particles can migrate into the tear film, resulting in increased debris within the lipid layer. This interferes with meibomian gland output, a key factor in maintaining tear film stability. Such mechanical obstruction and contamination can accelerate tear evaporation, leading to chronic irritation and inflammation (3, 39, 41, 42).

Moreover, ocular itching (38.4%), conjunctival hyperemia (36.2%), and eye pain (24.4%) were frequently reported. These symptoms may be explained by hypersensitivity reactions to preservatives, dyes, and fragrance components in cosmetic products (11, 26). Repeated exposure to such allergens may induce subclinical inflammation and exacerbate underlying allergic conjunctivitis or meibomitis. These findings were echoed in a similar cohort by Addis et al. (1), who found strong associations between poor label awareness and increased incidence of ocular allergic symptoms.

A notable 25% of users reported blurred vision, a concerning symptom suggestive of significant optical disturbance, possibly caused by a compromised tear film or transient epithelial disruption (39). Chemical irritation, reported by 15.7%, may reflect direct toxic effects of cosmetic ingredients or improper removal techniques involving aggressive cleansers (9, 14, 43).

The most severe complication reported was corneal abrasion, identified in 6.2% of users. Though infrequent, corneal abrasions represent a clinically serious outcome, with potential for infection, scarring, and permanent visual impairment. This aligns with findings by Wang and Craig (10), who emphasized that seemingly innocuous cosmetic practices can result in sight-threatening injuries when proper hygiene is not maintained (33).

Another frequently overlooked issue was madarosis (eyelash loss) reported in 43.7% of participants. Chronic use of mascara, adhesive false lashes, and aggressive removal techniques can exert mechanical traction on lash follicles. Kadri et al. (37) found a significant positive association between long-term cosmetic use and milphosis (eyelash loss), with 19% of medical students reporting eyelash loss. This observation was supported by **(author?)** (3), who linked chronic blepharitis, frequently exacerbated by mascara residues, to follicular damage and lash weakening (3).

While dry eye disease (DED) symptoms were prevalent in our cohort, objective diagnostic tests such as tear breakup time (TBUT) or Schirmer's test were not conducted. Ercan et al. (41) found no significant difference in TBUT among users and non-users, whereas other studies reported increased OSDI (Ocular Surface Disease Index) scores in habitual users (5, 6, 12, 41, 44, 45). Interestingly, this study found no significant difference in OSDI scores (*p* = 0.083), despite increased subjective discomfort (*P* < 0.001), highlighting the complex interplay between symptom perception and objective disease markers. This could partially explained by Alison Ng et al. (8) and Hunter et al. (39) who demonstrated that cosmetic use, particularly layering multiple products, may create a *cumulative* effect on ocular surface stress. This additive exposure to foreign substances increases the risk of subclinical inflammation, disruption of mucin layer integrity, and delayed epithelial healing—mechanisms central to the chronicity of cosmetic-induced ocular surface disease (20, 27, 34, 36, 46, 47).

## 5 Limitations

The study has few limitations that should be considered when interpreting or generalizing its findings. First, the study relied on self-reporting of adverse reactions (ARs), which may be subject to recall bias and potential under- or over-reporting, and the management of these ARs was not captured—as it was not part of the aims of the study—making it difficult to ascertain the true impact or appropriate treatments utilized by the participants. Secondly, the study did not account for the frequency with which participants applied eye makeup during the day—although daily use was considered indicative of regular usage. Lastly, the study—intentionally—did not gather data on specific brands or types of eye makeup, which precludes any assessment of causality between particular products and observed adverse effects.

## 6 Conclusions

This study highlights the extensive use of eye cosmetics among Jordanian women and indicates a high incidence of self-reported ocular adverse responses, which range from moderate irritation to clinically important consequences, including madarosis and corneal abrasion. Regular users tended to be younger, more educated, and professionally active, yet exhibited notable deficiencies in product literacy, hygiene practices, and cosmetic removal behaviors. Some of the risk factors we reported included young age, pre-existing allergies, and improper application or removal habits; these findings highlighted the need for public health education interventions, particularly among non-health-discipline populations.

The results reinforce the public health importance of cosmetovigilance and call attention to the underreported ocular risks of eye makeup use. Given the association between suboptimal practices and adverse reactions, the policymakers in general need to prioritize standardized product labeling, public awareness campaigns, and the incorporation of routine screening for cosmetic-related ocular symptoms in clinical settings. Future longitudinal and interventional research is needed to find causal relationships between specific product types and ocular adverse reactions and to inform the development of evidence-based guidelines for safer cosmetic use.

## Data Availability

The raw data supporting the conclusions of this article will be made available by the authors, without undue reservation.
